# Copper homeostasis and cuproptosis in health and disease

**DOI:** 10.1002/mco2.724

**Published:** 2024-09-17

**Authors:** Yunuo Yang, Jiaxuan Wu, Lisheng Wang, Guang Ji, Yanqi Dang

**Affiliations:** ^1^ Institute of Digestive Diseases China‐Canada Center of Research for Digestive Diseases Longhua Hospital Shanghai University of Traditional Chinese Medicine Shanghai China; ^2^ State Key Laboratory of Integration and Innovation of Classic Formula and Modern Chinese Medicine (Shanghai University of Traditional Chinese Medicine) Shanghai China; ^3^ Department of Biochemistry, Microbiology and Immunology, Faculty of Medicine University of Ottawa Ottawa Ontario Canada; ^4^ China‐Canada Centre of Research for Digestive Diseases University of Ottawa Ottawa Ontario Canada

**Keywords:** cancer, cardiovascular disease, copper homeostasis, cuproptosis, hereditary disease, liver disease, neurodegenerative disease

## Abstract

Copper is a vital trace element in human physiology, essential for the synthesis of numerous crucial metabolic enzymes and facilitation of various biological processes. Regulation of copper levels within a narrow range is imperative for maintaining metabolic homeostasis. Numerous studies have demonstrated the significant roles of copper homeostasis and cuproptosis in health and disease pathogenesis. However, a comprehensive and up‐to‐date systematic review in this domain remains absent. This review aims to consolidate recent advancements in understanding the roles of cuproptosis and copper homeostasis in health and disease, focusing on the underlying mechanisms and potential therapeutic interventions. Dysregulation of copper homeostasis, manifesting as either copper excess or deficiency, is implicated in the etiology of various diseases. Cuproptosis, a recently identified form of cell death, is characterized by intracellular copper overload. This phenomenon mediates a diverse array of evolutionary processes in organisms, spanning from health to disease, and is implicated in genetic disorders, liver diseases, neurodegenerative disorders, and various cancers. This review provides a comprehensive summary of the pathogenic mechanisms underlying cuproptosis and copper homeostasis, along with associated targeted therapeutic agents. Furthermore, it explores future research directions with the potential to yield significant advancements in disease treatment, health management, and disease prevention.

## Graphical abstract

Copper is a vital trace element in human physiology. Copper homeostasis and cuproptosis play important roles in health and disease development, including genetic disorders like Menkes disease and Wilson's disease, liver diseases such as nonalcoholic fatty liver disease and hepatocellular carcinoma, neurodegenerative disorders like Alzheimer's disease, amyotrophic lateral sclerosis, and Huntington's disease, as well as various cancers including colorectal, breast, lung, and pancreatic cancer, and cardiovascular diseases. This review encompasses both the mechanistic underpinnings and potential therapeutic interventions related to these processes.

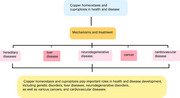

## 1 INTRODUCTION

Copper is a vital trace element within the human body, with the current recommended daily intake for adults ranging from 0.8 to 2.4 mg/day.^1^ The majority of copper is stored in muscle and bone (50–70%), with smaller amounts found in blood (5‐10%) and the liver containing the highest concentration among internal organs (approximately 20%). Copper plays a crucial role in various physiological processes such as energy metabolism, antioxidant defense, blood clotting, neurotransmitter synthesis, and iron metabolism.^1^, ^2^ Elevated concentrations of copper within the body can impede cellular homeostasis by perturbing redox equilibrium, resulting in cellular apoptosis.^3^ Conversely, insufficient levels of copper can hinder growth and immune function, potentially leading to the onset of various pathologies.

There has been a strong correlation between copper homeostasis and cuproptosis, as well as the progression of human health and disease. These processes are implicated in various evolutionary transitions from health to disease in organisms. This review systematically compiles and synthesizes the latest research findings on copper homeostasis and cuproptosis in the context of health and disease, examines their potential applications, and offers perspectives for future research directions.

Copper, being an essential trace element, plays a significant role in human health and disease. In the scientific community, copper homeostasis and cuproptosis have recently gained increasing attention due to the emergence of cuproptosis. The aim of this review is to provide an overview of copper homeostasis and cuproptosis, focusing on the mechanisms involved in maintaining copper homeostasis, particularly in the context of cuproptosis, including hereditary diseases, liver diseases, neurodegenerative diseases, cancers, and cardiovascular diseases. In addition to examining pertinent pharmaceuticals derived from these targets, this review will provide a comprehensive overview of the impact of copper homeostasis and cuproptosis on human health and disease, encompassing the underlying mechanisms and potential therapeutic approaches.

## 2 COPPER HOMEOSTASIS AND CUPROPTOSIS IN HEALTH

### 2.1 Copper homeostasis

#### 2.1.1 Historical background

As a transition metal with inherent redox capabilities, copper plays a critical role in biological processes, including the synthesis and activation of essential metabolic enzymes such as lysyl oxidase (LOX), cytochrome *c* oxidase (COX), and intracellular copper‐zinc superoxide dismutase (SOD1).^4^, ^5^ Copper, as an essential enzyme cofactor, plays a vital role in antioxidant processes, biological synthesis, bone formation, energy metabolism, and iron metabolism, among other physiological functions.^6^, ^7^

Studies have shown that dysregulation of copper homeostasis induces oxidative stress. Activation of Fenton‐like reactions by high copper concentration facilitates the formation of reactive oxygen species (ROS), consequently impairing cell morphology and enhancing lipid peroxidation. Furthermore, due to disruption of the ubiquitin–proteasome system, copper ions inhibit cellular protease activity and cell proliferation. Excessive copper ions can also trigger cuproptosis by promoting the aberrant oligomerization of lipoylated proteins. Additionally, copper is crucial for the maintenance of normal physiological cardiovascular function, and the copper transport protein 1 (CTR1) promotes the internalization through sulfinylation, thereby enhancing angiogenesis.^8^

Iron plays a crucial role in maintaining the body's physiological functions. Research suggests that copper can impact iron metabolism, particularly through its involvement in LOX and ceruloplasmin functions.^9^, ^10^ Deficiency in ceruloplasmin can disrupt iron metabolism and transportation, leading to abnormal iron accumulation in the body. Moreover, copper deficiency can manifest as hypochromic microcytic anemia, akin to iron deficiency.^11^, ^12^, ^13^ Furthermore, copper has been discovered to promote iron homeostasis within the body.^14^ Copper facilitates the release of iron from storage by promoting the biosynthesis of ceruloplasmin, an enzyme responsible for oxidizing iron in circulation.^9^ Copper is redistributed to tissues and organs involved in regulating iron homeostasis, including the intestines, liver, and blood, during episodes of iron deficiency.^9^ Arredondo et al.^15^ discovered that copper inhibits iron uptake in human colon adenocarcinoma cells, while conversely, iron inhibits copper uptake. The mechanism of iron uptake by the brain may undergo alterations in the presence of excess copper. Furthermore, a study has demonstrated that copper loading led to a reduction in iron uptake in relevant organs of rats.^16^ An animal experiment indicated that dietary copper deficiency resulted in iron‐deficiency anemia, potentially linked to decreased iron absorption and retention.^17^ Excessive copper overload suppresses the activity of divalent metal transporter 1, consequently impacting the intestine's absorption of iron.^9^, ^18^, ^19^

#### 2.1.2 Metabolism of copper

Copper is primarily absorbed into the human body through food intake. Foods rich in copper include oysters, scallops, pork liver, walnuts, oats, and buckwheat. In the small intestine, copper absorption primarily occurs, where Cu^2+^ is converted to Cu^1+^ by six‐transmembrane epithelial antigen of the prostate (STEAP, a metalloreductase). Afterward, it is taken up by the CTR1 in the epithelial cells of the small intestine. Copper then enters the bloodstream via the copper ion transport ATPase alpha peptide (ATP7A), which facilitates its distribution to all tissues throughout the body.^1^ Copper is mainly stored in the liver. The excess copper is mainly excreted into the intestines through bile and then eliminated in feces.^20^, ^21^, ^22^

Under normal physiological conditions, the content of copper within cells is maintained at a narrow range under the influence of a sophisticated network of proteins. This network comprises copper enzymes, copper chaperones, and membrane transporters, working in concert to coordinate the regulation of copper influx, efflux, and intracellular utilization.^23^ Copper enters cells and is shuttled to subcellular compartments with the aid of STEAP and CTR1. Within mitochondria, essential metabolic enzymes such as COX and SOD1 serve as critical components, pivotal for scavenging free radicals and safeguarding against oxidative damage.^24^ Copper serves as a vital cofactor for COX, essential for maintaining normal mitochondrial respiration.^25^ Copper initially binds to COX copper chaperone 17 (COX17), from where it is subsequently transported to COX through the action of COX11 and COX synthesis 1.^26^ On the other hand, the maturation of SOD1 relies on the insertion of copper ions,^27^ facilitated by copper superoxide dismutase chaperonin.^28^ Within the nucleus, copper participates in gene expression by interacting with transcription factors.^20^ Moreover, facilitated by the antioxidant 1 copper chaperone, copper is translocated to ATP7A and ATPase beta peptide (ATP7B) within the trans‐Golgi network, eventually reaching the lumen.^29^

#### 2.1.3 Introduction of copper oxidase and its physiological function

LOX enzymes are part of the secreted LOX family and are extracellular copper‐dependent enzymes.^30^ Studies indicate that various organs, including the heart, kidneys, lungs, liver, and lung, have been found to have profibrotic effects when these enzymes cross‐link collagen and elastin.^31^ Peptides derived from the N‐terminus of LOX may exhibit tumor‐suppressive properties.^32^ Furthermore, this enzyme family is implicated in cell proliferation, differentiation, and the development of cardiovascular diseases.^33^

COX serves as the terminal enzyme in the electron transport chain, functioning as both an oxygen acceptor and a rate‐limiting enzyme in mitochondrial respiration. It catalyzes the oxidation of cytochrome *c*, playing a crucial role in ATP generation.^34^, ^35^ Copper acts as a cofactor for the COX enzyme, being translocated to the mitochondria and subsequently inserted into the subunits COX1 and COX2, which are essential for maintaining the activity of enzyme.^36^

By disproportionating superoxide anion radicals, SOD1 scavenges ROS by promoting oxygen and hydrogen peroxide production, thus participating in the dynamic balance between oxygen and hydrogen peroxide.^37^ Moreover, research has demonstrated that SOD1 functions as a transcription factor in the regulation of gene expression and plays a role in metabolic pathways.^38^ The involvement of SOD1 in the pathogenesis of various diseases, such as familial amyotrophic lateral sclerosis (ALS), has been well documented.^38^ Additionally, studies have suggested a potential link between SOD1 and tumorigenesis, as elevated SOD1 levels are associated with poorer prognosis for various cancer types, such as breast and lung cancers.^39^ Furthermore, SOD1 has been implicated in the processes of aging and oxidative stress.^37^

### 2.2 Cuproptosis

#### 2.2.1 Core components and regulatory mechanisms of cuproptosis

Although copper is necessary for organism growth, it is important to consider the potential risks of copper overdose.^29^ In 2022, Tsvetkov et al.^23^ first described cuproptosis, a process in which intracellular copper accumulation leads to the binding of copper to thiooctylated components of the tricarboxylic acid cycle. This results in abnormal aggregation of lipoylated protein, disrupting mitochondrial respiration‐associated Fe−S cluster proteins, and ultimately leading to a proteotoxic stress response that results in cell death.^23^ Ferredoxin 1 (FDX1) functions as an upstream regulator of proteolipid acylation, with immunohistochemical staining revealing a strong correlation between FDX1 expression and lipoylated protein levels. Both FDX1 and proteolipid acylation play crucial roles in the regulation of cuproptosis.

The onset of cuproptosis is typically associated with distinct cellular morphological alterations. Animal studies have demonstrated that copper treatment induces mitochondrial contraction, chromatin rupture, endoplasmic reticulum damage, and cellular membrane rupture in zebrafish embryonic retinal cells.^40^

Mitochondria play a crucial role in the process of cuproptosis, which involves aerobic respiration and energy production in cells.^41^ Tsvetkov and colleagues^42^ observed that cells with a greater reliance on mitochondrial respiration exhibit heightened sensitivity to copper ion carriers compared with cells primarily undergoing glycolysis, such as embryonic retinal cells. This association has significant implications for understanding the mechanisms of cell death, particularly in the context of cuproptosis. Excessive copper accumulation has been shown to disrupt mitochondrial structure and function by enhancing membrane permeability and compromising membrane potential.^43^ Furthermore, mitochondrial glutathione has been demonstrated to inhibit cuproptosis by reducing thiooctylation and promoting dihydrolipoamide S‐acetyltransferase (DLAT) oligomerization.^44^

It is essential to regulate copper levels within a precise range in the body. When there is a dysregulation in the metabolic homeostasis of copper leading to abnormal levels of copper, it can result in the development of associated diseases.

## 3 COPPER HOMEOSTASIS AND CUPROPTOSIS IN CANCER

**FIGURE 1:**
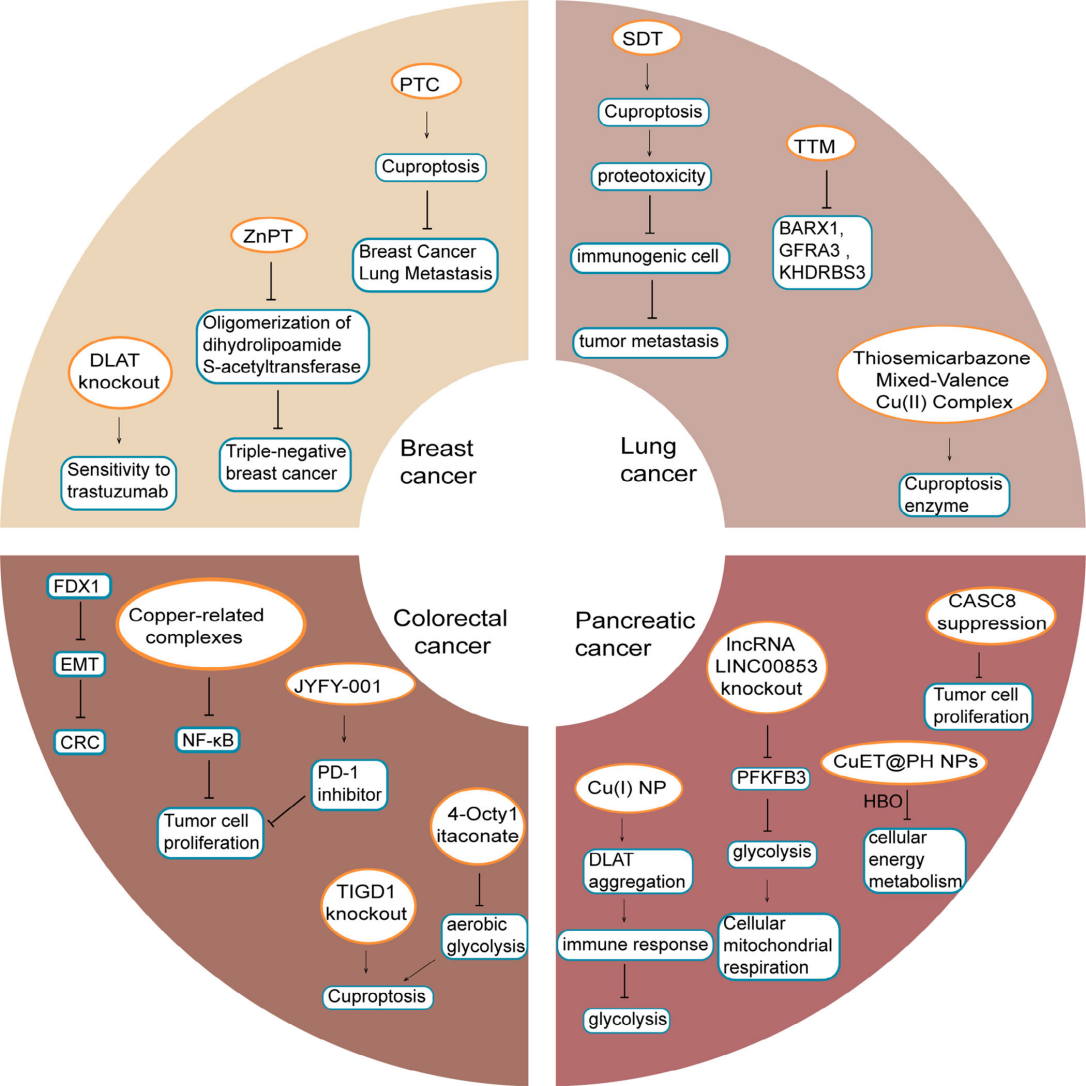
Copper homeostasis and cuproptosis in cancer. In colorectal cancer (CRC), copper‐related complexes have been shown to impede tumor cell proliferation by inhibiting the NF‐κB pathway in colorectal cancer cell lines. Additionally, ferredoxin 1 (FDX1) has been found to hinder the growth and advancement of CRC by suppressing epithelial–mesenchymal transition (EMT). Furthermore, JYFY‐001 has been demonstrated to enhance the antitumor effects of programmed cell death protein 1 (PD‐1) inhibitors. Moreover, 4‐octyl itaconate inhibits aerobic glycolysis by specifically targeting glyceraldehyde‐3‐phosphate dehydrogenase, thereby facilitating cuproptosis. Knockdown of tigger transposable element derived 1 (TIGD1) has been observed to potentially enhance cuproptosis in CRC cells. In breast cancer, a platelet vesicle (PV)‐coated cuprous oxide nanoparticle (Cu_2_O)/TBP‐2 cuproptosis sensitization system (PTC) has been shown to inhibit metastasis by targeting and inducing cuproptosis. Additionally, knockdown of DLAT has been found to increase breast cancer sensitivity to trastuzumab. Zinc pyrithione hinders the advancement of triple‐negative breast cancer by stimulating the oligomerization of dihydrolipoamide S‐acetyltransferase. In the context of lung cancer, thiotetramide has the potential to counteract the increased expression of BarH‐like homeobox 1 (BARX1), GDNF family receptor alpha 3 (GFRA3), and KH domain containing RNA binding, signal transduction associated 2 (KHDRBS2). Elevated levels of copper in vivo lead to cuproptosis, a process that is intensified by SDT resulting in proteotoxicity, which leads to the destruction of immunogenic cells and the suppression of tumor metastasis. Thiosemicarbonyl mixed‐valent copper(II) complexes have the ability to eliminate lung cancer cells by activating various pathways, including cuproptosis enzymes. In pancreatic cancer, the inhibition of cancer susceptibility candidate 8 (CASC8) impacts the proliferation of pancreatic cancer cells. The Cu(I) nanoparticles induce the aggregation of dihydrolipoamide S‐acetyltransferase (DLAT), resulting in cuproptosis, while also enhancing immune responses and suppressing tumor growth. Copper nanodrug dopamine and hydroxyethyl starch‐stabilized copper diethyldithiocarbamate nanoparticles (CuET@PH NPs) effectively suppress energy metabolism in pancreatic ductal adenocarcinoma stem cells under hyperbaric oxygen conditions. Knockdown of the cuproptosis‐associated long noncoding RNA LINC00853 inhibits glycolysis and enhances cellular mitochondrial respiration by reducing the expression of the glycolytic enzyme glycolytic kinase 6‐phosphofructo‐2‐kinase/fructose‐2,6‐biphosphatase 3 (PFKFB3).

Cuproptosis has been linked to tumor development, prompting increased research interest in the relationship between cuproptosis and cancer. Analysis of pan‐cancer multiomics and single‐cell sequencing data has revealed significant expression of cuproptosis‐associated genes across various tumor types, with cyclin dependent kinase inhibitor 2A exhibiting the highest mutation frequency (49%) among all tumors. The study found a negative correlation between cuproptosis scores and tumor microenvironment scores, but a positive correlation with PD‐L1 expression. Additionally, cuproptosis was associated with poor tumor prognosis^45^ (Figure 1).

### 3.1 Colorectal cancer

Colorectal cancer (CRC), a prevalent malignancy in the gastrointestinal tract, is influenced by various unfavorable risk factors including obesity, population aging, smoking, and lack of physical activity.^46^ In terms of global incidence and mortality, CRC is the third most prevalent cancer worldwide,^47^ constantly threatening human public health safety.

Colorectoscopy remains the preferred method for diagnosing CRC, yet its semi‐invasive nature often leads to low patient compliance and limited clinical implementation, resulting in delayed diagnoses in many cases. Recent advancements in molecular biology technology have expanded the options for detecting and treating CRC. Copper‐mediated cuproptosis and its associated proteins serve as valuable indicators for the diagnosis and prognosis of CRC.

Studies have recently demonstrated that serum copper levels are associated with CRC. One study revealed that blood copper levels exceeding 930 µg/L were linked to a heightened risk of developing CRC, suggesting that blood copper levels could serve as a potential indicator for early detection of the disease.^48^ Conversely, an examination of 4663 participants found no statistically significant correlation between serum copper levels and CRC.^49^ Furthermore, a meta‐analysis encompassing 26 studies indicated that individuals with CRC exhibited higher average copper levels compared with controls, although this disparity did not reach statistical significance.^50^

Copper‐related complexes inhibit tumor cell proliferation by inhibiting the nuclear factor kappa‐B (NF‐κB) pathway.^51^ Shao et al.^52^ examined the association between the expression of cuproptosis‐related molecules and CRC, finding that the levels of cuproptosis‐related molecules were linked to tumor node metastasis (TNM) stage, pathological characteristics, tumor immune microenvironment, and drug resistance in CRC patients. Specifically, high DLAT expression in CRC patients was associated with chemotherapy and immunotherapy resistance, but also correlated with improved prognosis and could serve as a potential biomarker for CRC.^52^ Research findings suggest that DLAT is linked to immune‐related pathways, the tumor microenvironment, and various immune cell infiltrations.^53^ Additionally, Li et al.^54^ conducted an analysis on cuproptosis‐related characteristics in CRC and established a risk score prediction model consisting of seven copper‐related genes (CRGs, including dipeptidyl peptidase 7, G protein‐coupled receptor associated sorting protein 1, unc‐5 netrin receptor C, RAB3B, the member of RAS oncogene family, protocadherin 9, solute carrier family 18 member A2, cerebellar degeneration related protein 2 like). Patients with a high risk score exhibited a shorter overall survival and lower complete remission rate.^54^ Furthermore, Gong et al.^55^ developed a predictive model for CRC focusing on cuproptosis and disulfidptosis, which demonstrated strong predictive ability. Zhu et al.^56^ utilized a principal component analysis algorithm to develop a cuproptosis score, which showed that patients with a lower cuproptosis score demonstrated increased survival, immune cell activation, and tumor purity. Additionally, FDX1 was found to have a negative correlation with tumor invasion in colon adenocarcinoma according to database analysis.^57^ Furthermore, FDX1 was shown to impede the growth and progression of CRC by inhibiting epithelial‐mesenchymal transition.^58^ Wu et al.^59^ further confirmed that knockdown of tigger transposable element derived 1 may enhance cuproptosis in CRC cells. 4‐Octyl itaconate has been shown to inhibit aerobic glycolysis by targeting glyceraldehyde‐3‐phosphate dehydrogenase, leading to the promotion of cuproptosis and its potential role as an anti‐CRC agent.^60^ Additionally, the upregulation of the cuproptosis‐related gene miR‐653 in CRC tissues has been linked to increased cell proliferation and decreased apoptosis through negative regulation of dihydrolipoamide dehydrogenase (DLD) expression.^61^ Furthermore, Shi et al.^62^ have introduced a novel copper chelator, JYFY‐001, which demonstrates efficacy in inhibiting tumor cell proliferation, inducing apoptosis, and enhancing the antitumor effects of programmed cell death protein 1 (PD‐1) inhibitors, while also exhibiting a favorable safety profile.

### 3.2 Breast cancer

Breast cancer is a prevalent malignancy affecting the epithelium of the breast or ductal epithelium. It can be categorized into three subtypes: Hormone positive, human epidermal growth factorreceptor 2(HER2)MYC proto‐oncogene, bHLH transcription factor positive, and triple‐negative breast cancer.^63^ The global incidence of breast cancer is one in 20 individuals, with a higher prevalence of one in eight individuals in high‐income countries.^64^ Risk assessment models have been developed to estimate an individual's likelihood of developing breast cancer, and advancements in technology have enhanced the accuracy of these predictive models. Sha et al.^65^ developed a nomogram to predict survival probability in triple‐negative breast cancer through analysis of databases. Patients with low CRG‐scores exhibited elevated tumor mutational load and immune activation, correlating with favorable survival outcomes.^65^

Cuproptosis‐associated long noncoding RNAs (lncRNAs) may play a role in breast cancer regulation. Researchers utilize six cuproptosis‐associated lncRNAs to construct a comprehensive model for prognostic prediction and therapy sensitivity assessment in breast cancer patients, aiding in clinical treatment decision‐making.^66^ Immunohistochemical analysis of breast cancer surgical specimens, coupled with bioinformatics analysis, demonstrated a significant correlation between MYC proto‐oncogene, bHLH transcription factor(c‐Myc)AIE photosensitizer expression and the stemness phenotype, as well as copper‐induced cytotoxicity, in breast cancer tissues. Furthermore, the presence of protumor cuproptosis biomarkers was found to be associated with tumor cell stemness.^67^ Utilizing a database, researchers identified solute carrier family 31 member 1 (SLC31A1) as a potential cuproptosis‐related gene that is upregulated in breast cancer and is linked to poor patient prognosis.^68^

Through extensive investigation of cuproptosis‐related targets, a range of novel drugs have been developed. Ning et al.^69^ developed a platelet vesicle‐coated cuprous oxide nanoparticle/AIE photosensitizer(TBP‐2)lipoylated protein cuproptosis system that effectively hinders lung metastasis of breast cancer by selectively targeting and inducing cuproptosis in tumor cells, while concurrently enhancing central memory T cells in peripheral blood and preventing tumor reinvasion. Additionally, the tumor suppressor p53 plays a crucial role in regulating the formation of iron‐sulfur clusters and the copper chelator glutathione, thereby modulating intracellular cuproptosis levels in a bidirectional manner.^70^ Subsequent investigations have identified LINC01614 as a potential upstream regulator of SLC31A1.^71^ A comprehensive analysis across multiple cancer types indicates that diminished expression of copper‐related genes ATP7B and DLAT is linked to enhanced immune response activation, improved patient survival rates, and heightened responsiveness to chemotherapy.^72^ Specifically, DLAT has been identified as a factor contributing to resistance against targeted therapeutic interventions. Suppression of DLAT expression has been shown to heighten the sensitivity of breast cancer cells to trastuzumab, with the gene playing a role in the regulation of organelle fission, chromosome segregation, nuclear division, hormone‐mediated signaling pathways, and chromosome condensation.^73^ Zinc pyrithione has been shown to impede the advancement of triple‐negative breast cancer by perturbing copper homeostasis through the facilitation of DLAT oligomerization, a key indicator of cuproptosis.^74^

### 3.3 Lung cancer

Lung cancer, arising from the tracheal, bronchial mucosal, or glandular tissues, is the most prevalent malignant neoplasm affecting the lung. Histopathologically, it is predominantly categorized into non‐small cell carcinoma (comprising adenocarcinoma and squamous carcinoma) and small cell carcinoma.^75^ Based on statistical data, lung cancer constitutes 11.6% of all cancer cases and is the predominant form of cancer, leading to the highest mortality rates attributed to cancer. Factors such as pollution and smoking are frequently cited as common contributors to the development of lung cancer.^76^ Approximately 2 million new cases of lung cancer are reported annually, resulting in a mortality range of 10−76 million deaths.^77^

Zhang et al.^78^ utilized database analysis to identify five oncogenic driver genes associated with lung cancer, including CD79b molecule, phosphatidylethanolamine binding protein 1, protein tyrosine kinase 2 beta, syntaxin binding protein 1, and zinc finger protein 671, and subsequently developed a proportional hazard regression model. The study results indicated a notable decrease in survival rate among the high‐risk group.^78^ Chen et al.^79^ developed a gene signature for lung adenocarcinoma (LUAD) and observed elevated expression of BarH‐like homeobox 1, GDNF family receptor alpha 3, and KH domain containing, RNA binding, signal transduction associated 2 in an in vitro cuproptosis model. Additionally, they found that the copper chelator tetrathiomolybdate ammonium could reverse this up‐regulation.^79^ Xie et al.^80^ created a gene score to predict overall survival, revealing that patients with low CRG scores had increased tumor mutational load, enhanced immunoreactivity, and a greater likelihood of survival. Yang et al.^81^ developed a prognostic signature utilizing eight cuproptosis‐associated genes to forecast overall survival in patients with LUAD, and subsequently illustrated the impact of secreted phosphoprotein 1 on the proliferation, migration, and stemness of LUAD cells. Wang et al.^82^ devised a nomogram incorporating ten cuproptosis‐associated lncRNAs. Varied risk categories exhibit distinct sensitivities to conventional therapeutic modalities for lung cancer, thereby offering valuable guidance for the selection of diverse treatment strategies in clinical practice.^82^ Ma et al.^83^ identified nine lncRNAs associated with immune checkpoints in LUAD. The key cuproptosis gene pyruvate dehydrogenase E1 component subunit alpha (PDHA1), which is crucial for cuproptosis, plays a role in altering glucose metabolism. High levels of PDHA1 in LUAD patients are linked to a negative prognosis.^84^ Researchers developed prognostic scores for non‐small cell lung cancer using CRGs, revealing that the high‐risk group with high prognostic scores had worse outcomes, increased tumor mutational load, and reduced immune infiltration compared with those with high cuproptosis scores.^85^ The study revealed a correlation between cuproptosis and ferroptosis in LUAD, with genomic analyses illustrating the interplay between copper and iron oxidase regulators. This information was utilized to develop the Cu‐Fe score, a tool for predicting treatment outcomes with various therapies.^74^ Additionally, sorafenib and erastin, known inducers of ferroptosis, were found to inhibit FDX1 degradation, enhance the aggregation of copper‐dependent lipoylated proteins, and stimulate cuproptosis in primary hepatocellular carcinoma (HCC) cells.^86^ In vivo and in vitro studies have demonstrated that thiosemicarbonyl mixed‐valent copper (II) complexes have the ability to induce cell death in lung cancer cells through the activation of multiple pathways, including cuproptosis enzymes.^87^ More recently, researchers have developed copper‐substituted zinc–aluminum ternary layered double hydroxide nanosheets for the combined treatment of lung cancer metastasis using acoustic‐dynamic therapy (SDT). This treatment involves the use of excess copper in vivo to trigger cuproptosis, which is further enhanced by SDT to induce proteotoxicity, leading to the elimination of immunogenic cells and the inhibition of tumor metastasis.^88^

### 3.4 Pancreatic cancer

Pancreatic cancer, a malignancy arising from the epithelial and follicular cells of the pancreatic ducts, ranks as the seventh most common cause of cancer‐related mortality globally.^89^ The incidence of pancreatic cancer has increased by over twofold in the past quarter‐century due to demographic shifts and lifestyle modifications. Risk factors for the disease encompass advanced age, tobacco use, obesity, diabetes, alcohol consumption, and chronic pancreatic injury.^90^ Timely detection of tumors is imperative for a favorable prognosis; however, over 80% of patients are in advanced stages of the disease, resulting in a 5‐year survival rate and prognosis that is notably poor.^89^ Consequently, it is crucial to promptly evaluate patient prognosis and determine suitable treatment modalities. Recent research has implicated copper in the pathogenesis of pancreatic cancer, suggesting that cuproptosis may serve as a valuable tool for prognostic assessment in patients. The identification of cuproptosis‐related characteristics utilizing five lncRNAs has the potential to serve as a prognostic tool for individuals with pancreatic cancer. Among these lncRNAs, cancer susceptibility 8 exhibits heightened expression levels and influences the immune microenvironment of tumors. Subsequent investigations have revealed that inhibiting cancer susceptibility candidate 8 impacts the proliferation, apoptosis, migration, and chemokine expression in pancreatic cancer cells.^91^ Sun et al.^92^ developed a prognostic signature for pancreatic adenocarcinoma comprising four immune‐related genes to forecast patient outcomes and immune profiles, thereby enhancing the ability to tailor personalized combination therapies centered on immunotherapy. In the context of pancreatic cancer, the downregulation of cuproptosis‐associated lncRNA LINC00853 hinders glycolysis and enhances cellular mitochondrial respiration through the reduction of glycolytic kinase.^93^ Furthermore, DLAT expression is correlated with immune cell infiltration, making it a potentially valuable prognostic and immunological biomarker for pancreatic adenocarcinoma. Some researchers have constructed risk assessment models with high predictive accuracy based on DLAT expression levels.^94^

Recently, researchers have introduced several innovative drugs aimed at targeting cuproptosis. Yu et al. have developed cascade nanosystems that, when administered to tumor cells, release copper ions to induce cuproptosis, thereby supporting chemokinetic therapy to augment the efficacy of combination treatments.^95^ Copper complex nanoparticles (Cu(I) NP) have also been shown to trigger the release of copper complexes, disrupt mitochondrial function, facilitate the aggregation of DLAT, ultimately resulting in cuproptosis, as well as eliciting immune responses and impeding tumor progression.^96^ An investigator recently published a study detailing the use of an acoustic wave‐triggered cascade lactate depletion strategy with a semiconductor polymer nanoreactor for cuproptosis immunotherapy in pancreatic cancer. This approach utilizes Cu^+^ to activate antitumor immune effects through cuproptosis, resulting in the death of immunogenic cells.^97^ Additionally, copper nanodrug dopamine and hydroxyethyl starch‐stabilized copper diethyldithiocarbamate nanoparticles (CuET@PH NPs) were developed based on cuproptosis, effectively inhibiting energy metabolism in pancreatic ductal adenocarcinoma stem cells under hyperbaric oxygen conditions.^98^ Numerous studies have elucidated the intricate relationship between copper homeostasis and various types of cancers. The correlation between copper levels, particularly cuproptosis, and diverse cancers has been established, with current research efforts concentrated on investigating the association between CRGs and cancer progression, prognosis, and drug resistance through data analysis and the development of predictive models. However, there remains a lack of conclusive biological evidence regarding the specific mechanisms through which these genes contribute to disease development, necessitating further exploration.

## 4 COPPER HOMEOSTASIS AND CUPROPTOSIS IN NEURODEGENERATIVE DISEASE

**FIGURE 2:**
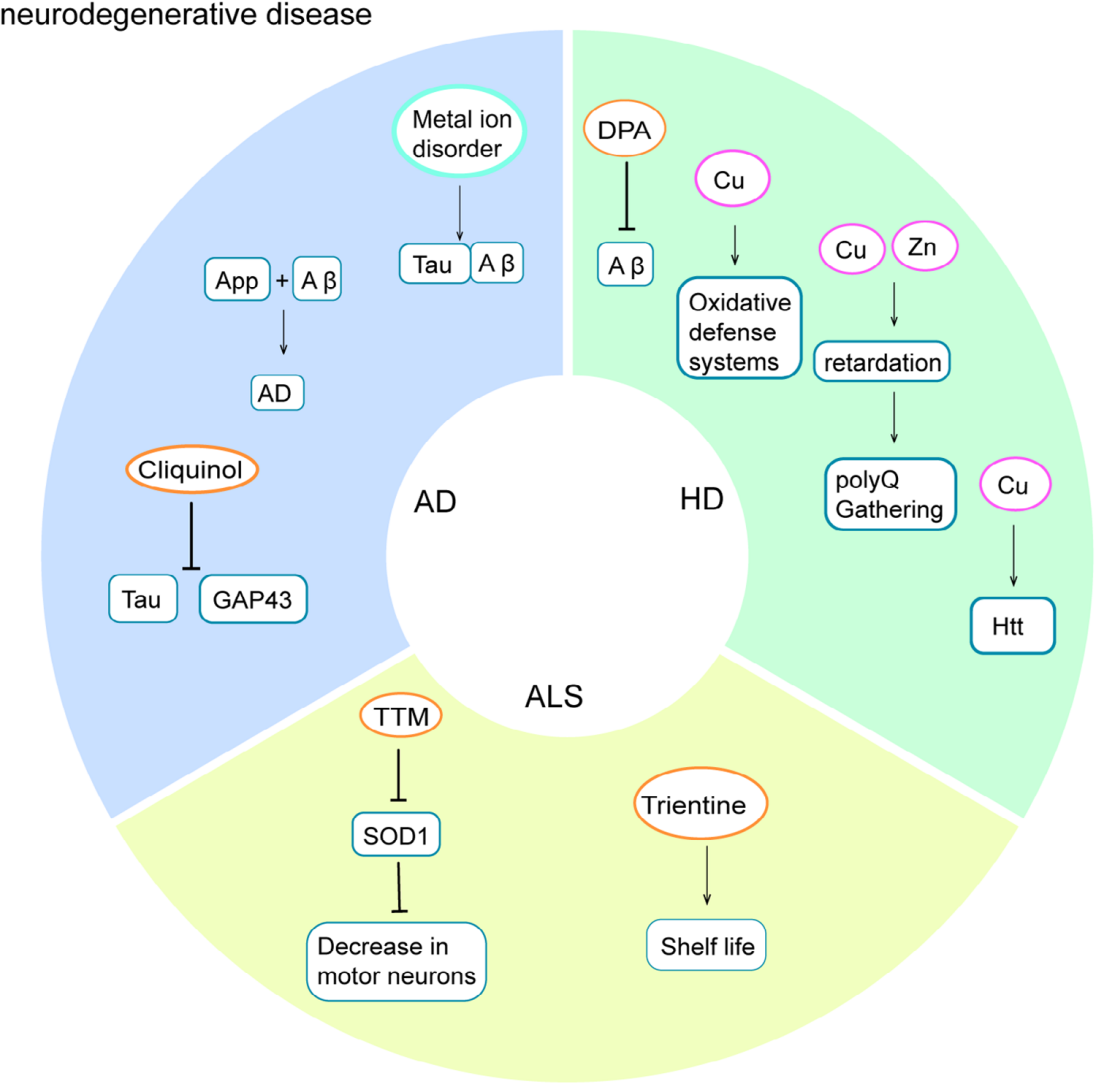
Copper homeostasis and cuproptosis in neurodegenerative disease. In Alzheimer's disease (AD), disruption of metal ion homeostasis contributes to the accumulation of β‐amyloid (Aβ) and tau proteins. The interaction between the copper‐binding domain of amyloid precursor protein (APP) and specific amino acid residues of Aβ may play a role in the pathogenesis of AD. Treatment with the copper chelator clioquinol has been shown to decrease levels of Tau protein and growth‐associated protein. In amyotrophic lateral sclerosis (ALS), treatment with tetrathiomolybdate ammonium (TTM) has been found to reduce the formation of superoxide dismutase (SOD1) aggregates and mitigate the loss of motor neurons. Administering trientine prior to disease onset significantly extends the lifespan of ALS mice. In Huntington's disease (HD), d‐penicillamine (DPA) has been shown to decrease the aggregation of β‐amyloid in a drosophila model of HD. Copper has been found to enhance the formation of thioflavin S‐positive β‐amyloid structures within the huntingtin (HTT) protein aggregates and to activate the antioxidant defense system. Prolonged exposure to copper, zinc, and their combinations has been shown to lead to the inhibition and aggregation of polyglutamine (polyQ) in both muscle and neuronal tissues.

There are several neurodegenerative diseases associated with copper homeostasis and cuproptosis, such as Alzheimer's disease (AD), ALS, and Huntington's chorea (HD)^99^ (Figure 2).

### 4.1 Alzheimer's disease

AD, a degenerative disorder of the central nervous system marked by progressive cognitive decline and behavioral impairment, is the most prevalent form of dementia.^100^ The prevalence of AD is projected to increase twofold in Europe and threefold globally by the middle of the century.^101^ Despite the lack of consensus on the etiology of AD, several hypotheses have been proposed to elucidate its pathogenesis. The initial theory posited in the study is the amyloid cascade hypothesis, which suggests that the production of β‐amyloid (Aβ) through the hydrolysis of amyloid precursor protein by β‐ and γ‐secretase proteins leads to an increased ratio of hydrophobic Aβ1‐42/43. This accumulation of Aβ in the brain results in the formation of age spots and subsequently triggers inflammatory reactions, neurotoxicity, and a series of pathological processes. These processes further enhance the deposition of Aβ, creating a cascade of amplified responses.^102^ The subsequent hypothesis involves the abnormal phosphorylation of tau proteins. The tau protein, a microtubule‐associated protein, plays a crucial role in maintaining cytoskeletal stability. Abnormal phosphorylation of tau protein results in the formation of double‐stranded helical filaments and neuronal fiber tangles, ultimately leading to microtubule collapse and neuronal death. Furthermore, various theories such as the cholinergic theory, oxidative stress hypothesis, and neuroinflammation hypothesis have been proposed to explain the pathogenesis of AD.^103^, ^104^

Copper metabolism disorders have also been linked to the progression of AD. A bibliometric analysis of 73 studies revealed alterations in copper, iron, zinc, and selenium levels in AD patients.^105^ A meta‐analysis indicates a reduction in copper levels in the brains of patients with AD and an elevation in serum/plasma levels, correlating with an augmented risk of AD.^106^ Recent research has identified disruptions in metal ion homeostasis, particularly copper, in vulnerable brain regions of individuals with AD, facilitating the aggregation of Aβ and its interaction with tau. The excessive buildup of copper additionally fosters cuproptosis.^103^ The direct interaction between the copper‐binding domain of amyloid precursor protein and various amino acid residues of Aβ may contribute to the pathogenesis of AD.^107^ A study involving 20 patients diagnosed with AD demonstrated that the copper chelator Clioquinol effectively decreased levels of tau protein and growth‐associated protein by day 21.^108^

### 4.2 Amyotrophic lateral sclerosis

ALS is a chronic and progressive neurological disorder characterized by symptoms of skeletal muscle weakness, atrophy, fasciculations, and medullary paralysis, ultimately leading to respiratory failure and death within approximately three years.^109^ The exact cause of ALS remains uncertain, with potential factors including genetic predisposition, abnormal immune responses, lifestyle choices, and metabolic dysregulation. Despite significant research efforts, the current diagnosis and treatment of ALS continue to be less than ideal.^110^ Recent advancements in molecular biology have highlighted the importance of targeted therapies for maintaining copper homeostasis.

It has been proposed that alterations in the SOD1 gene are crucial in the onset of ALS, as abnormalities in this gene may cause mitochondrial issues, harm motor neurons, and eventually result in fatality.^111^ Variants of SOD1 have been shown to impact copper homeostasis in various organisms. Animal experiments demonstrated that the copper chelator tetrathiomolybdate ammonium effectively decreased the presence of SOD1 aggregates, mitigated the decline of motor neurons and axons, reduced skeletal muscle atrophy in mice, and extended their survival time.^112^ Additionally, a study observed diminished copper levels in the cerebrospinal fluid of ALS patients compared with control subjects.^113^ Furthermore, when SOD1 is not bound to zinc ions, copper ions in SOD1 facilitate the generation of superoxide anions and subsequent production of peroxynitrite through reaction with nitric oxide.^114^ Recent research has provided evidence of a correlation between cuproptosis and the onset of ALS. Swindell et al.^115^ conducted a study that identified differentially expressed genes in ALS through peripheral blood analysis, highlighting the strong association between the gene encoding the superoxide dismutase copper chaperonin and survival rates. Similarly, Jia et al.^116^ identified five key predictor genes of ALS (BRCA1 associated deubiquitinase 1, SMG1 nonsense mediated mRNA decay associated PI3K related kinase, BCL2 associated transcription factor 1, DEAH‐box helicase 15, eukaryotic translation initiation factor 4 gamma 2) through the analysis of peripheral blood gene expression data using a machine learning model, and successfully developed an support vector machine prediction model with promising results in evaluation. Solovyev et al.^117^ identified copper reduction as a distinctive characteristic of ALS through the examination of cerebrospinal fluid. Animal studies have demonstrated that the copper chelator trientine enhances survival in ALS mice.^118^ Furthermore, the combination of high doses of trientine and ascorbic acid administered prior to disease onset significantly extended the survival of ALS mice and prevented the deterioration of motor function.^119^ Edaravone is currently a primary medication used in the clinical management of ALS, with LeBlanc et al.^120^ developing 17 Edaravone analogues that exhibit potent copper‐chelating abilities.

### 4.3 Huntington disease

HD is a progressive neurodegenerative disorder characterized by dominant polyglutamine amplification at the N‐terminus of the huntingtin (HTT) protein.^121^ Potential interventions for HD encompass various therapeutic approaches targeting Huntington's DNA and RNA, protein clearance, DNA repair pathways, inflammation, and cellular replacement. However, currently available therapies lack specificity for HD.^122^

Drosophila experiments have demonstrated a correlation between the expression levels of copper metabolism‐related genes and the progression of HD. The decrease in copper levels resulted in a significant reduction in the presence of oligomeric and aggregated HTT.^121^ Copper has been shown to enhance the formation of thioflavin S‐positive Aβ structures within HTT aggregates and impact autophagy levels in the brain. The use of the d‐penicillamine (DPA), demonstrated a significant decrease in Aβ aggregation in a Drosophila HD model.^123^ Prolonged exposure to copper, zinc, and their combinations has been found to impair locomotor function and cause developmental delays, leading to the aggregation of polyglutamine in both muscle and nerve cells, ultimately resulting in neurodegeneration. On the contrary, rutin demonstrates chelating capabilities in the HD model and serves a protective function in neurodegenerative lesions.^124^ Pfalzer et al.^125^ noted an increase in copper levels in cerebrospinal fluid samples at the onset of HD. A comprehensive examination indicates a decrease in copper levels in HD brain tissue.^126^ Administration of copper orally was observed to enhance the antioxidant defense mechanism in the rat model with HD, potentially leading to a deceleration of the progression of the disease.^127^ Moreover, studies have shown a disruption in copper ion homeostasis in AD, specifically in relation to the proteins Aβ and tau. The gene SOD1 has been identified as playing a crucial role in ALS, with variations in this gene impacting motor neuron function through modulation of copper homeostasis. Furthermore, researchers have identified other genes such as superoxide dismutase copper chaperonin as differentially expressed in these diseases and have developed predictive models for disease progression. Genes involved in copper metabolism have been found to impact levels of HTT, with copper chelators such as DPA and rutin showing potential for improving symptoms of HD.

## 5 COPPER HOMEOSTASIS AND CUPROPTOSIS IN LIVER DISEASE

The liver serves as the principal repository for copper in the human body and is integral to copper metabolism. Imbalances in copper levels can lead to oxidative stress, disruption of proteasome function, and impaired angiogenesis, ultimately resulting in cellular damage and the progression of liver disease.^4^

### 5.1 Hepatocellular carcinoma

HCC ranks as the fifth most prevalent cancer worldwide and stands as the second leading cause of cancer‐related mortality.^128^ In recent years, there has been a notable increase in the global incidence of HCC, presenting significant burdens and complexities to the field of global public health.^129^ Major risk factors for HCC encompass hepatitis B, hepatitis C, aflatoxin exposure, and alcohol consumption.^130^ Additionally, emerging research suggests potential links between metabolic syndrome, nonalcoholic steatohepatitis (NASH), and the development of HCC.^131^

There is already evidence of metabolic disturbances in copper in HCC patients. In a study with 175 HCC patients, serum copper levels exhibited a positive correlation with alanine aminotransferase and the Barcelona stage of HCC.^132^ Methylation patterns of CRGs are observed to be differentially upregulated or downregulated in HCC, potentially linked to CRG activation.^133^ Gene set variation analysis suggests that CRGs might modulate cuproptosis via metabolic pathways, thereby impacting HCC development.^134^ By prompting DLAT expression and activating the PI3K/mTOR, maternal embryonic leucine zipper kinase enhances mitochondrial function and facilitates HCC.^135^ Proteolipid acylation and FDX1 and are essential regulatory genes involved in cuproptosis. Bioinformatics analysis reveals that FDX1 levels are significantly reduced in HCC compared with normal tissues. A higher cuproptosis‐related risk score, derived from FDX1, is correlated with shorter patient survival.^136^

Yan et al.^137^ developed a prognostic risk model utilizing CRGs (glycine cleavage system protein H, lipoyltransferase 1, and cyclin dependent kinase inhibitor 2A), revealing that downregulation of lipoyltransferase 1 can impede the proliferation and invasion of HCC cells. A prognostic signature associated with cuproptosis (Cu‐PS), comprising MAGE family member A6, cytochrome P450, family 26, subfamily b, polypeptide 1, complement C7, erythropoietin, and hexokinase 2, has been identified and analyzed, revealing a significant positive correlation with tumor node metastasis classification, drug sensitivity, tumor mutation load, and response to immunotherapy in HCC. Patients with elevated Cu‐PS exhibit a poorer prognosis, particularly those in advanced TNM stages compared with early stages.^138^ Chen et al.^139^ established a cuproptosis‐related signature involving five CRGs‐solute carrier family 25 member 28, pyridoxal kinase (PDXK), C‐type lectin domain family 3 member B, hepsin, and ring finger protein, transmembrane 1 and observed significantly upregulated expression of PDXK in HCC tissues. Deficiency of PDXK inhibits the proliferation, migration, and invasion of HCC cells.^139^ CRGs can modulate HCC development by influencing the tumor immune microenvironment and immune checkpoints. Key genes prion protein, synuclein alpha, and COX17 impact multiple immune cell infiltrations and are notably associated with cytotoxic T lymphocyte antigen 4 and PD‐1 levels. Higher expression of prion protein and synuclein alpha fosters immune cell infiltration in HCC with a poorer prognosis, while COX17 suppresses HCC immune cell infiltration.^140^ Additionally, cuproptosis‐associated lncRNA is closely linked to HCC and serves as a prognostic indicator. The researchers developed a risk assessment model based on HLA complex group 15, AC105020.5, AC079209.1, and AC019069.1, which can be utilized to evaluate the prognosis of HCC.^141^

**FIGURE 3:**
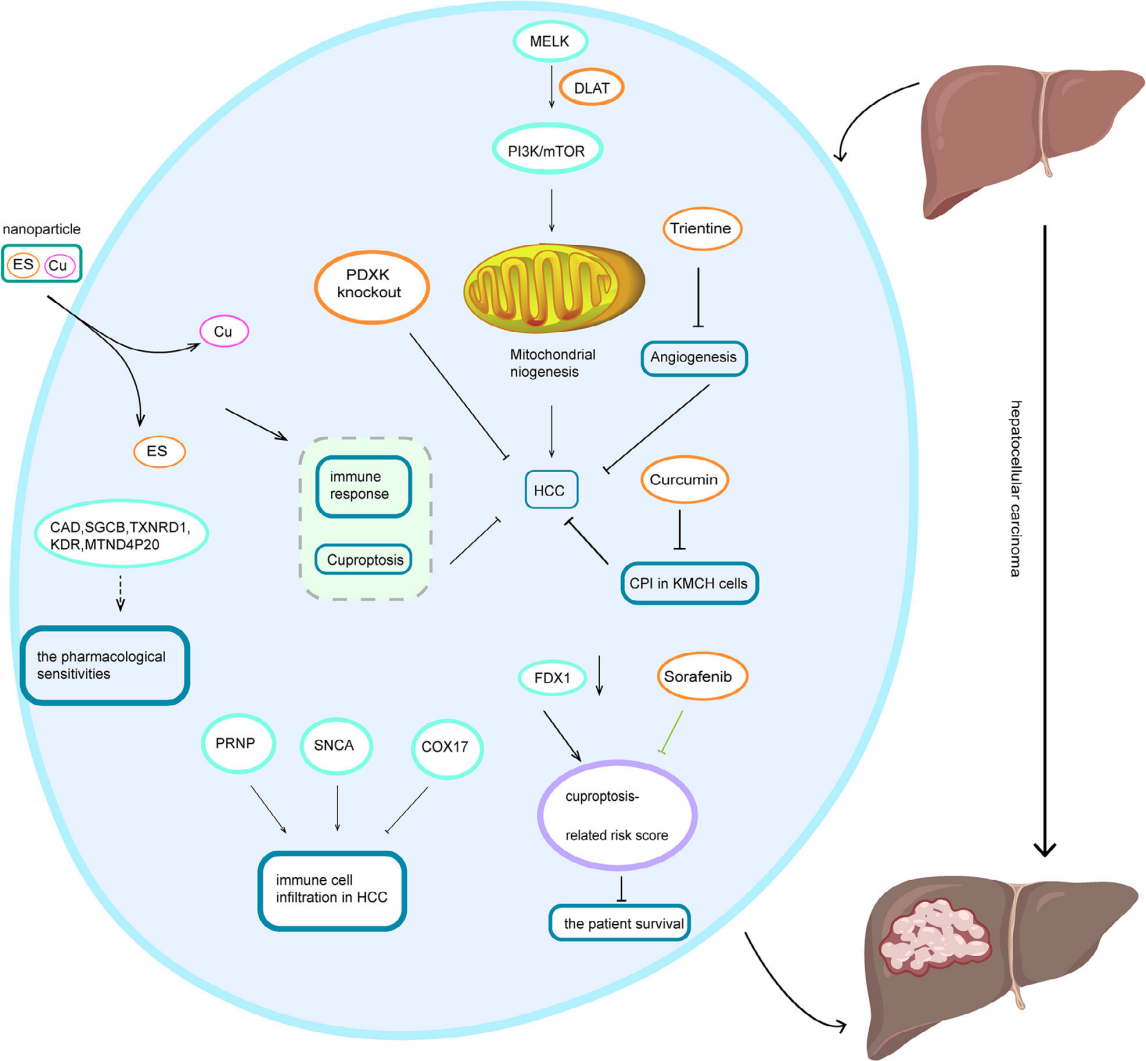
Copper homeostasis and cuproptosis in hepatocellular carcinoma. Copper‐related genes (CRGs) influence immune cell infiltration. Maternal embryonic leucine zipper kinase (MELK) stabilizes mitochondrial function by activating PI3K/mTOR signaling. Decreased ferredoxin 1 (FDX1) level raises cuproptosis‐related risk score, and sorafenib suppresses the score. Curcumin downregulates cuproptosis potential index. Knockdown of pyridoxal kinase (PDXK) inhibits proliferation, migration, and invasion of hepatocellular carcinoma (HCC) cells. Studies have encapsulated Cu and ES with ROS‐sensitive polymers, which induces cuproptosis and activates immune response.

Sorafenib is the standard treatment for advanced HCC,^142^ and patients responsive to treatment exhibit a decrease in cuproptosis‐related risk scores.^136^ Elesclomol (ES), a copper ion carrier, serves as an inducer of cuproptosis. Studies have encapsulated copper and ES within ROS‐sensitive polymers to formulate nanomedicines. Following the translocation into cancer cells, it is released upon ROS induction. ES subsequently triggers cuproptosis and activates the immune response.^143^ Angiogenesis represents a critical hallmark of tumorigenesis, including HCC.^144^, ^145^ The copper chelator trientine has been found to inhibit angiogenesis and induce apoptosis of tumor cells, thereby impeding tumor progression.^146^ Curcumin potentially inhibits HCC development by reducing the cuproptosis potential index.^147^ Furthermore, a risk‐prognostic model of CRGs in HCC patients, composed of caudal, sarcoglycan beta, thioredoxin reductase 1, kinase insert domain receptor, and MT‐ND4 pseudogene 20, has been established. Patients with high and low scores on this model demonstrate varying sensitivities to different therapeutic drugs for HCC. This observation implies a potential correlation between the expression of CRGs and pharmacological sensitivities in HCC, thereby presenting novel therapeutic strategies for the disease.^148^ Emerging evidence indicates that cuproptosis is associated with the progression and prognosis of HCC; however, the precise mechanisms underlying the involvement of cuproptosis in HCC pathogenesis necessitate further investigation (Figure 3).

### 5.2 Nonalcoholic fatty liver disease

Nonalcoholic fatty liver disease (NAFLD) is a clinicopathological syndrome characterized by the excessive accumulation of fat in hepatocytes, excluding cases attributable to alcohol consumption and other well‐defined hepatotoxic factors. It is strongly associated with obesity, insulin resistance, type 2 diabetes mellitus, and dyslipidemia.^149^, ^150^, ^151^ NAFLD has the potential to progress to NASH, which can ultimately lead to cirrhosis and HCC. The global prevalence of NAFLD is approximately 25%, with rates exceeding 30% in South America.^149^ NAFLD frequently coexists with other metabolic disorders, presenting a significant public health challenge as its prevalence rises.^152^, ^153^

**FIGURE 4:**
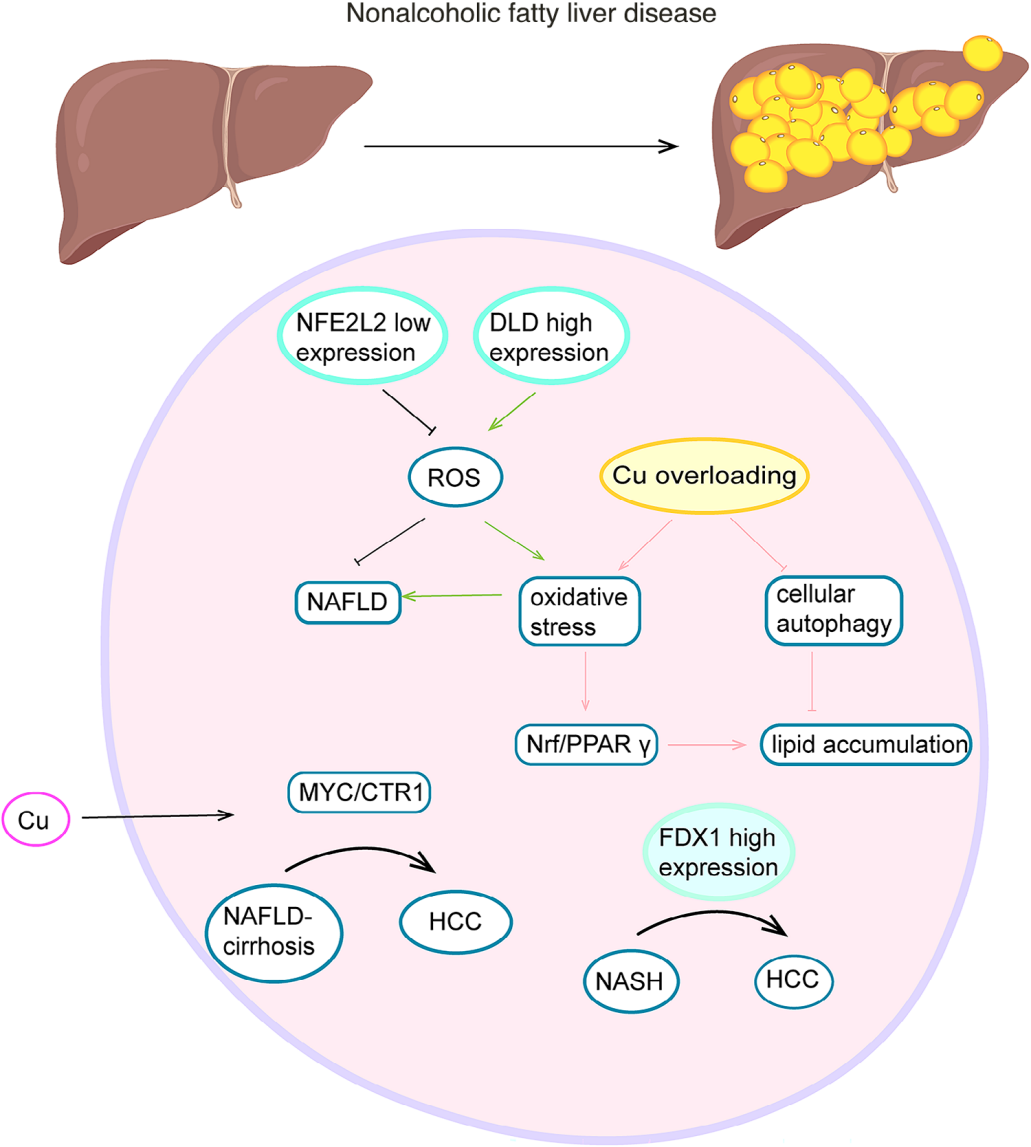
Copper homeostasis and cuproptosis in NAFLD. NFE2 like bZIP transcription factor 2 (NFE2L2) and dihydrolipoamide dehydrogenase (DLD) modulate disease progression through the regulation of reactive oxygen species (ROS) levels. Copper overload influences lipid accumulation, while elevated serum copper levels and ferredoxin 1 (FDX1) promote the transition from nonalcoholic fatty liver disease to hepatocellular carcinoma.

Porcu et al.^154^ observed significantly elevated serum copper levels in patients with NAFLD‐cirrhosis, with further increases as the disease advanced to HCC. Mechanistically, heightened serum copper levels regulate the MYC/CTR1 axis, thereby promoting tumor growth, migration, and invasion. CRGs such as NFE2 like bZIP transcription factor 2, DLD, DNA polymerase delta 1, and pyruvate dehydrogenase E1 subunit beta has been found to be potential biomarkers for NAFLD diagnosis.^155^ DNA polymerase delta 1 is upregulated in NAFLD and correlates with the cytokine‐cytokine receptor interaction pathway. NFE2 like bZIP transcription factor 2 mitigates NAFLD by reducing ROS production, while DLD, highly expressed in NAFLD, enhances ROS generation, exacerbating oxidative stress and NAFLD progression.^156^ Additionally, studies have demonstrated high expression of FDX1 in NASH, playing a pivotal role in NASH‐HCC development and providing a new promising therapeutic target for the disease.^157^ Copper overload promotes oxidative stress and cellular autophagy, with oxidative stress activating the NFE2 like bZIP transcription factor 2/peroxisome proliferator activated receptor gamma (NRF2/PPARγ) pathway and autophagy mitigating copper‐induced lipid deposition, thus offering protection against copper‐induced NAFLD.^158^, ^159^ However, activation of the NRF2/PPARγ pathway can induce transcription of targeted genes and promote adipogenesis.^160^, ^161^ Altered copper levels are associated with the course of NAFLD, but there is still a lack of research on how much of a regulatory role cuproptosis plays in NAFLD and its specific mechanisms (Figure 4).

## 6 COPPER HOMEOSTASIS AND CUPROPTOSIS IN HEREDITARY DISEASES

**FIGURE 5:**
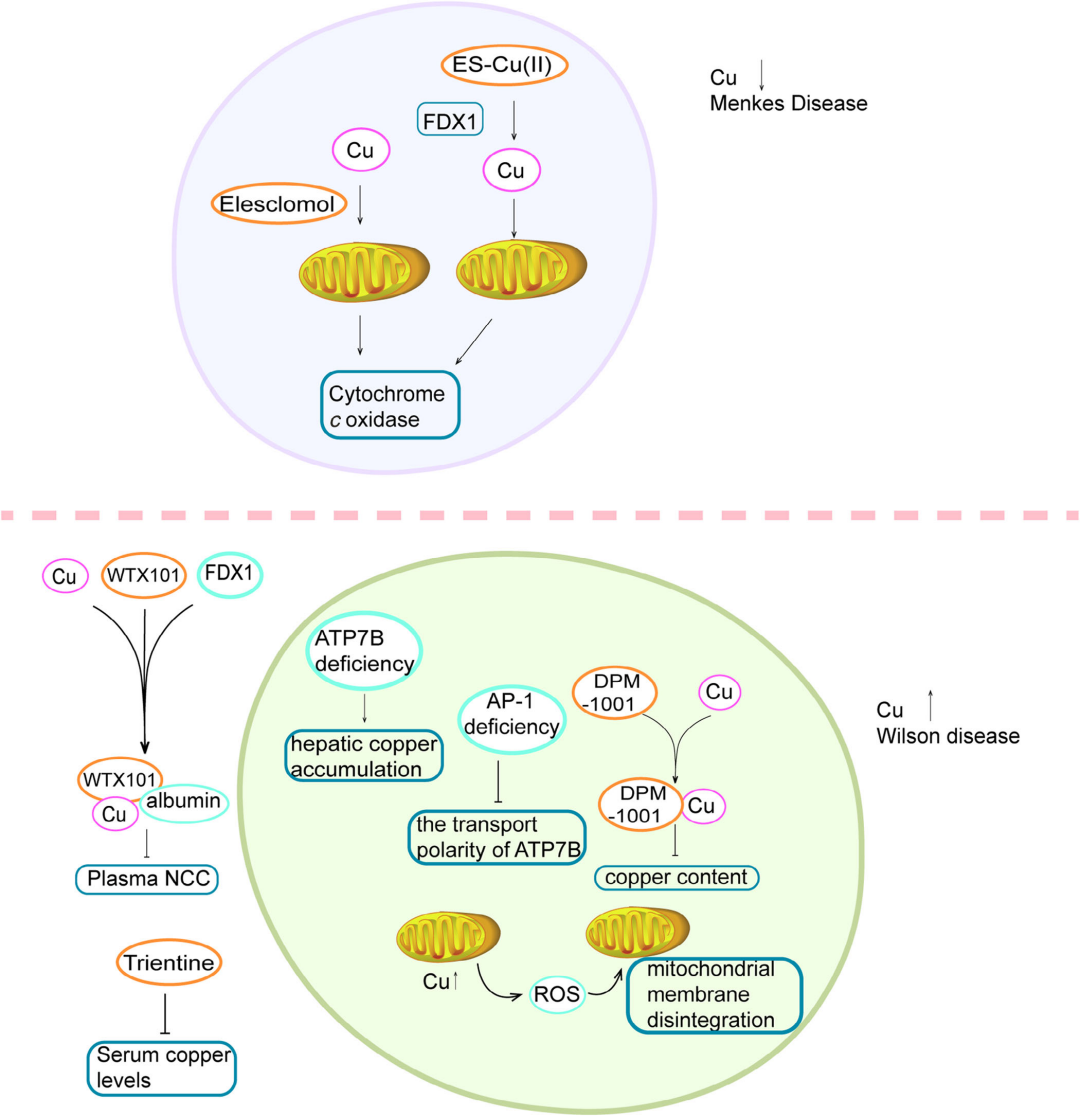
Copper homeostasis and cuproptosis in hereditary diseases. In Wilson disease, the deficiency of the adaptor protein (AP‐1) hinders the activity of ATPase beta peptide (ATP7B), leading to the accumulation of copper in the liver. Both DMP‐1001 and trientine are effective in reducing copper levels. In Menkes disease, the transport of copper to mitochondria, facilitated by the small molecule elesclomol (ES), enhances the expression of cytochrome *c* oxidase in the brain. The reduction of ES‐Cu(II) to Cu(I) by ferredoxin 1 (FDX1) catalyzes the metallation of the copper enzyme cytochrome *c* oxidase (COX) within the mitochondria.

Recent research has elucidated the link between cuproptosis and inherited disorders such as Menkes disease (MD), attributed to copper deficiency,^162^ and Wilson's disease (WD),^163^, ^164^ characterized by abnormal copper accumulation. Both conditions are classified as copper storage disorders, emphasizing the importance of restoring copper balance in treatment strategies (Figure 5).

### 6.1 Menkes disease

MD is characterized by copper deficiency due to abnormal copper metabolism.^165^ The prevalence of MD among live‐born boys has been estimated to be one in 35,000.^166^ MD is caused by mutations in the ATP7A gene, which disrupts copper homeostasis in nerve tissue, liver, and blood, leading to impaired function of copper‐dependent enzymes such as COX, dopamine β‐hydroxylase, lysine oxidase, and tyrosinase.^167^, ^168^ These disruptions in enzyme function contribute to the clinical manifestations of the disease, such as stunted growth, psychomotor developmental disorders, spasticity seizures, and various hair abnormalities. A recent study has demonstrated the potential therapeutic effects of the copper‐delivering small molecule compound ES in the treatment of MD.^169^ Animal studies have shown that ES can improve pathological changes and reduce lethality in Menkes model mice.^170^ Furthermore, these studies have indicated that ES facilitates copper transport to mitochondria, leading to increased expression of COX in the brain and mitigation of neurodegenerative lesions.^171^ The reduction of ES‐Cu(II) to Cu(I) by mitochondrial matrix reductase FDX1 catalyzes the release of copper into the mitochondria, promoting metallation of COX.^172^ Animal experiments demonstrated that in lactating and juvenile copper‐injected chimeric mutants, the CTR1 protein translocated from the apical membrane to the cytoplasm of proximal renal tubular epithelial cells, thereby inhibiting copper transport from the primary urine and providing protection against copper toxicity.^173^ Additionally, pleckstrin homology domain containing A5 (PLEKHA5), PLEKHA6, and PLEKHA7 recruit PDZ domain containing 11 to distinct plasma membrane locations, facilitating ATP7A targeting to the cell periphery in high copper environments.^174^ Furthermore, the use of crystalline nanostructured metal–organic framework material allows for the controlled release of copper, leading to a reduction in neuronal degeneration and disease progression.^175^

### 6.2 Wilson's disease

WD is a rare condition, with an estimated carrier frequency ranging from one in 90 to 150 individuals and an incidence of approximately one in 30,000.^176^ Patients with a diagnosis of WD may present with a range of liver‐related conditions, including acute liver failure, chronic hepatitis, cirrhosis, and Coombs‐negative hemolytic anemia.^176^ Liver damage is typically the primary and prevalent manifestation of WD.^163^, ^177^ The accumulation of copper in the liver in WD leads to the release of nonfibrin‐bound copper into the bloodstream, leading to systemic copper accumulation in organs such as the brain, eyes, and kidneys. Ultimately, this can result in cirrhosis, damage to the basal ganglia, keratoconus, and kidney injury.^178^ WD is a hereditary disorder of copper metabolism caused by mutations in the ATP7B gene.^179^, ^180^ These mutations result in impaired copper metabolism, as ATP7B is highly expressed in liver tissues. Mutations in ATP7B underlie the pathogenesis of WD, disrupting copper homeostasis.^181^, ^182^ Hepatocyte‐specific ATP7B deficiency promotes hepatic steatosis and obesity, with hepatic copper accumulation and increased urinary copper excretion.^183^ ATP7B, a copper‐detoxifying ATPase, demonstrates apical or luminal trafficking polarity. Deficiency of the adaptor protein alters the trafficking polarity of ATP7B in response to intracellular copper levels, suggesting potential implications for the diagnosis and treatment of WD.^184^ Proper regulation of copper levels is essential for cellular mitochondrial function, with normal mitochondrial respiration relying on sufficient copper. Excessive hepatic copper levels can negatively impact mitochondrial function, as mitochondria are especially vulnerable to copper overload.^25^ Alterations in mitochondrial structure are among the early manifestations of WD.^28^ Excessive copper accumulation within mitochondria leads to ROS generation, culminating in mitochondrial membrane disruption and hepatocyte death.^185^

Copper chelators, as cuproptosis reducers, are currently used in the clinical treatment of WD. The main copper chelators that have been entered into clinical studies or approved for treatment of WD include DPA, trientine, bis‐choline tetrathiomolybdate. DPA is an United States Food and Drug Administration (US FDA)‐approved first‐line treatment for WD in the United States; however, approximately one‐third of patients experience intolerance to this drug. In 2022, the US FDA approved trientine as an alternative treatment for WD in DPA‐intolerant individuals of all ages. A recent study found that trientine was equally as effective as DPA in reducing serum copper levels, with no reported adverse events. Copper chelators, such as trientine, play a crucial role in the clinical management of WD by reducing cuproptosis.^186^ Bis‐choline tetrathiomolybdate targets intracellular hepatocytic copper and reduces nonceruloplasmin‐bound copper by forming a complex with albumin, facilitating copper excretion via bile.^187^ A phase 2 clinical trial involving 28 WD patients showed a success rate of 71% with Bis‐choline tetrathiomolybdate treatment over 24 weeks; however, 40% of patients experienced liver enzyme elevations.^187^ Despite their efficacy, these copper chelators are associated with toxic side effects and necessitate lifelong administration, posing challenges to patient compliance. Consequently, efforts are underway to develop novel copper chelators to broaden WD treatment options and reduce serum copper levels. Chel2 promotes fecal copper excretion in WD mouse models.^188^ DPM‐1001 exhibits high copper‐binding specificity, enhancing cell survival and reducing copper levels, thereby ameliorating WD symptoms.^189^ Additionally, studies have demonstrated that methanobactin reverses hepatic injury during acute copper accumulation in the livers of ATP7B‐deficient rat.^190^ Methanobactin SB2 facilitates excess hepatic copper excretion via the biliary pathway.^191^ Current research has clarified the pathogenesis of WD, which is caused by excessive accumulation of copper in the body, and the development of drugs targeting cuproptosis has made some progress and has been applied to clinical treatments.

## 7 COPPER HOMEOSTASIS AND CUPROPTOSIS IN CARDIOVASCULAR DISEASE

**FIGURE 6:**
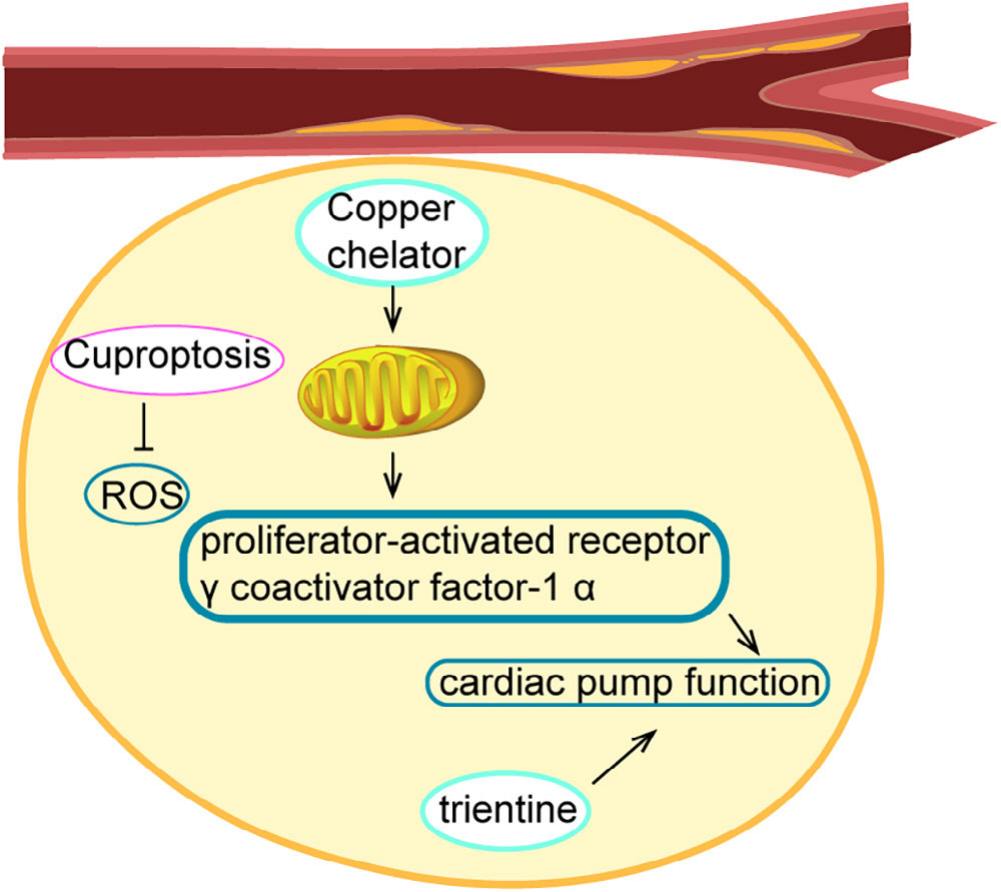
Copper homeostasis and cuproptosis in cardiovascular disease. Cuproptosis enhances the accumulation of reactive oxygen species (ROS) in the myocardium. Treatment with a copper chelator has been shown to repair rat myocardial mitochondria and enhance the function of proliferator‐activated receptor γ coactivator factor‐1 α, ultimately restoring cardiac pumping function. Additionally, trientine has been found to improve cardiac pump function.

Research indicates that disruptions in copper homeostasis, specifically in the regulation of copper chaperones or transporters, as well as copper deficiency, play a significant role in the development of various heart diseases.^192^ Copper influences heart disease through its impact on mitochondrial function, while dysregulation of copper homeostasis affects FDX1 and lipoic acid synthetase, leading to copper‐induced mortality.^193^ Cuproptosis is implicated in the pathogenesis of cardiovascular diseases such as atherosclerosis, heart failure, and dilated cardiomyopathy by disrupting lipid balance and promoting oxidative stress, mitochondrial impairment, and dysfunction of endothelial cells^194^ (Figure 6).

In the context of atherosclerosis, the upregulation of FDX1 and SLC31A1, along with the downregulation of glutaminase, was observed.^49^ FDX1 directly binds to copper, thereby inducing proteotoxic stress, while SLC31A1 facilitates the uptake of copper from the circulation into cells. Glutaminase, on the other hand, enhances intracellular glutathione synthesis to counteract oxidative stress.^195^ Wang et al.^196^ identified five CRGs and developed a diagnostic model with high diagnostic accuracy based on these findings. Furthermore, two distinct molecular subgroups were identified in atherosclerosis, exhibiting significant differences in mitochondrial outer membrane permeability and primary immunodeficiency.^196^ Chen et al.^197^ discovered upregulation of SLC31A1 and SLC31A2 expression, as well as downregulation of SOD1 in atherosclerotic plaques, suggesting their potential utility as diagnostic biomarkers for atherosclerosis. Cuproptosis, a process that promotes the accumulation of ROS, is closely linked to the aging process. Elevated levels of copper ions in aging populations have been shown to contribute to vascular senescence and cardiovascular disease.^198^ Researchers have developed chiral/phase dual‐engineered nanoenzymes that enrich copper and iron atoms in a low‐oxidation state when exposed to aging conditions, leading to the onset of cuproptosis. The d‐chiral platinum–palladium–copper–iron nanoenzymes have been found to exhibit antiaging and antiatherosclerotic effects in various aging models.^199^ Chen et al.^200^ formulated a risk assessment model incorporating ten CRGs in congestive heart failure, revealing a significant association between regulatory T cells and the expression levels of pivotal cuproptosis genes such as FDX1, DLD, DLAT, pyruvate dehydrogenase E1 subunit beta, and PDHA1. Subsequently, through rigorous clustering analysis, four key genes (hydroxysteroid dehydrogenase like 2, beta‐carotene oxygenase 2, corin, serine peptidase, and small nucleolar RNA, H/ACA box 80A) were identified in diabetes‐related heart failure, demonstrating their role in modulating cuproptosis in cardiomyocytes of patients by influencing immune system functionality.^201^ Elevated levels of advanced glycosylation end products in diabetic cardiomyopathy contribute to intracellular copper accumulation and the upregulation of the activating transcription factor 3/spi‐1 proto‐oncogene/SLC31A1 axis, thereby promoting cuproptosis.^202^ A study examining the relationship between immune cells and cardiomyopathy‐related genes in dilated cardiomyopathy revealed a significant positive association between eosinophils and SLC31A1, while DLD exhibited a negative correlation with monocytes.^203^ Additionally, six signature genes (septin 1, C‐type lectin domain containing 11A, ISG15 ubiquitin like modifier, prolyl 3‐hydroxylase 3, serine dehydratase like, and inka box actin regulator 1) linked to immune infiltration were identified as potential diagnostic biomarkers for dilated cardiomyopathy.^204^ Several studies have demonstrated the potential of copper chelators to repair rat myocardial mitochondria, enhance myocardial function, and modulate the activity of proliferator‐activated receptor γ coactivator factor‐1 α, thereby restoring cardiac pumping efficiency.^205^ Animal research has also indicated that trientine can enhance cardiac pump function and normalize levels of myocardial copper, copper chaperones, and synthesis of COX1.^206^

## 8 CONCLUSION AND PROSPECTS

As one of the trace elements in the human body, copper is essential for the production of various important enzymes, which play pivotal roles in energy production, iron metabolism, antioxidant defense, and the maintenance of normal nerve function.^29^ Copper is absorbed through the small intestine and subsequently stored in the liver. Its levels are intricately regulated by a complex network of proteins to maintain a narrow range. Cuproptosis is a novel cell death due to copper overaccumulation.

Perturbations in copper levels have been strongly linked to tumorigenesis, with numerous scholars validating the association between cuproptosis‐related genes and various types of cancer, including colorectal, lung, breast, and pancreatic cancers, and developing predictive models based on these findings. Various antitumor drugs aimed at targeting copper homeostasis are currently under development.

Copper mediates the progression of neurodegenerative diseases, and there have been studies confirming the alteration of copper levels in AD, ALS and HD. The key genes have been identified to predict disease progression, and relevant drugs are currently in the process of development.

Given the liver's role as the primary storage organ for copper, the relationship between copper homeostasis and the incidence and progression of liver diseases such as HCC and NAFLD is significant. The levels of CRGs are intricately connected to the pathogenesis of HCC and NAFLD. The use of a risk assessment model based on CRGs can aid in evaluating disease progression, prognosis, and drug responsiveness. Various drugs targeting cuproptosis have been investigated for the treatment of HCC, including sorafenib, ES, and trientine. However, the precise mechanism by which cuproptosis contributes to HCC development requires further elucidation for validation. Furthermore, the existing body of research on copper and NAFLD has predominantly examined the implications of copper deficiency, with a limited number of studies exploring the concept of cuproptosis, which involves excessive copper accumulation, in the development of NAFLD. The majority of current investigations have concentrated on the impact of intracellular copper levels on liver function.

The most pertinent hereditary disorders associated with copper homeostasis are MD and WD, resulting from copper deficiency and excess, respectively. MD is caused by mutations in the ATP7A gene, leading to progressive neurological deterioration and eventual fatality. Treatment options for MD include copper supplementation and supportive care, while advances in gene technology have deepened the study of MD pathogenesis, and the development of CRGs provides new targets for disease treatment. Copper supplementation can elevate copper levels in the body, while copper salt formulations like ES and MOF‐74 (Cu) are being developed. WD is a result of impaired copper metabolism due to ATP7B mutations, leading to mitochondrial ultrastructure disruption from copper overload. Current clinical management of WD relies on copper chelators therapy with agents like DPA, bis‐choline tetrathiomolybdate, and trientine, which are linked to adverse effects. Novel copper chelators under development, including Chel2, DPM‐1001, and methanobactin SB2, seek to mitigate these concerns.

Additionally, copper imbalance and cuproptosis play a role in the pathogenesis of cardiovascular disease, with research indicating a significant association between copper levels and cardiovascular morbidity and mortality. Therefore, restoring copper homeostasis is considered a crucial strategy in the treatment of cardiovascular disease.

Currently, many drugs for metabolic diseases have been developed targeting copper homeostasis and cuproptosis (Table 1).

**TABLE 1:** Related drugs targeting copper homeostasis or cuproptosis.

Name	Classification	Diseases	Research and development phase	References
d‐Penicillamine	Copper chelator	WD	US/China approved	^186^
Bis‐choline tetrathiomolybdate	Copper chelator	WD	Phase 3 clinical trial	^187^
Chel2	Copper chelator	WD	Preclinical studies	^188^
DPM‐1001	Copper chelator	WD	Preclinical studies	^189^
Methanobactin	Copper chelator	WD	Preclinical studies	^190^
Sorafenib	Ferroptosis inducer	Advanced HCC	US/EU approved	^142^
Elesclomol	Copper ion carriers	Solid tumor, MD	Phase 1 clinical trial	^143^
Trientine	Copper chelator	HCC, ALS	Preclinical studies	^146^
Clioquinol	Copper chelator	AD	Preclinical studies	^108^
Rutin	Copper chelator	HD	Preclinical studies	^124^
JYFY‐001	Copper chelator	CRC	Preclinical studies	^62^
Zinc pyrithione	Copper chelator	Breast cancer	Preclinical studies	^74^

Research on cuproptosis and its connection to copper homeostasis and disease has advanced significantly and holds promise for further investigation. However, studies investigating the relationship between cuproptosis and various diseases, such as hereditary diseases, liver diseases, neurodegenerative diseases, cancers, and cardiovascular diseases, are still in their nascent stages. The majority of these studies primarily involve correlational analyses between cuproptosis‐related genes and disease traits. Nonetheless, there is a notable absence of conclusive evidence to elucidate the regulatory mechanisms of cuproptosis. The complex nature of cuproptosis mechanisms, combined with the lack of definitive biomarkers for monitoring its activity, poses obstacles in the development of inhibitors or inducers for the treatment of liver disease. Additionally, the impact of these interventions on copper levels in the human body is not well understood, leading to apprehensions regarding potential toxicities. Copper homeostasis and cuproptosis are critical factors in the pathogenesis of numerous diseases, prompting further investigation into the therapeutic potential of targeting copper.

In conclusion, while cuproptosis represents a promising area of research as a recently identified mode of cell death in both physiological and pathological contexts, elucidating its precise mechanisms requires additional study and comprehension.

## AUTHOR CONTRIBUTIONS

*Design, and supervision; revising of original draft*: Yanqi Dang. *Design, and supervision; manuscript editing and revision*: Guang Ji. *Writing of original draft*: Yunuo Yang. *Manuscript editing and revision*: Jiaxuan Wu and Lisheng Wang. The final manuscript has been reviewed and approved by all the authors.

## CONFLICT OF INTEREST STATEMENT

All the authors declare no conflict of interest.

## ETHICS STATEMENT

Not applicable.

## Data Availability

Not applicable.
